# Efficacy and safety of dupilumab in two adolescents with severe atopic dermatitis

**DOI:** 10.31744/einstein_journal/2021RC6064

**Published:** 2021-04-28

**Authors:** Mara Giavina-Bianchi, Pedro Giavina-Bianchi

**Affiliations:** 1 São PauloSP Brazil Dermatologista, São Paulo, SP, Brazil.; 2 Universidade de São Paulo Faculdade de Medicina São PauloSP Brazil Faculdade de Medicina, Universidade de São Paulo, São Paulo, SP, Brazil.

**Keywords:** Dermatitis, atopic/drug therapy, Conjunctivitis, allergic, Immunosuppressive agents, Severity of illness index, Dermatologic agents, Dupilumab, Azathioprine, Adolescent, Efficacy, Safety

## Abstract

We report the cases of two adolescent siblings with severe atopic dermatitis, who, despite weighing approximately 40kg, presented a good response to dupilumab with the off-label dose for individuals aged 12 years and weighing 60kg. Both had already used cyclosporine, azathioprine, methotrexate and oral corticosteroids for long periods, plus topical treatments with no adequate disease control. Skin lesions were constant and widespread, with frequent skin infections and very poor quality of life, with numerous physical and psychosocial consequences, such as dropping out of school activities due to severe itching, appearance and bullying. They also showed delayed growth and development. In 2018, dupilumab, an immunobiological agent, was approved for treatment of moderate to severe atopic dermatitis in adults and, in 2019, extended to the 12-17-year age group. Although it had already been approved by the Brazilian Health Surveillance Agency, the 200mg presentation (indicated for the weight of patients) was not available, with no expected arrival date. Therefore, weighing the risks and benefits of the situation of both, we chose to treat them with an adult dose (loading dose of 600mg subcutaneously, and 300mg subcutaneously every 2 weeks) despite the low weight. So far, they have received eight injections, showing significant improvement of disease and quality of life. There were no major adverse effects, only worsening of allergic conjunctivitis in one of them. The patients and their family are very satisfied, and we believe that the therapy has been successful.

## INTRODUCTION

Atopic dermatitis (AD) is a chronic, recurrent, extremely pruritic, inflammatory disease of the skin, with desquamative, erythematous maculopapular or vesicular lesions, accompanied by dryness, crusting, and/or lichenification. Secondary infection by viruses or bacteria is common. The prevalence is 15% in children and 5% in adults, and has been increasing.^(^^1^^–^^3^^)^

Living with AD can be difficult, especially for those who require prolonged systemic treatment, since the drugs present significant toxicity. Intense itching and skin lesions can cause sleep disturbances, anxiety, depression, and low self-esteem, affecting the quality of life of patients and families.^(^^4^^)^

Pathogenesis of AD includes modification of the skin barrier, which may be associated with mutations in the filaggrin gene, increased colonization by *Staphylococcus aureus*, and exacerbated Th2 immune response, with sensitization to allergens, elevated levels of immunoglobulin E (IgE), and eosinophilia in the blood. Cyclosporine, mycophenolate mofetil, azathioprine, and methotrexate are the most commonly used immunosuppressive treatments.^(^^4^^–^^7^^)^ New therapies, such as dupilumab and Janus kinase enzyme (JAK) inhibitors, based on the pathogenesis of AD, are more effective and less harmful, and have been changing the approach to moderate to severe AD.^(^^5^^)^ Currently, dupilumab is approved for moderate to severe AD, severe asthma, and chronic rhinosinusitis with nasal polyposis in children 6 years and older, adolescents 12 years and older, and adults 18 years and older, respectively.^(^^8^^)^

The following report describes the great improvement in treatment of severe AD in the first two adolescents, weighing less than 60kg, treated in Brazil with dupilumab.

The study was approved by the Research Ethics Committee of *Hospital Israelita Albert Einstein* (HIAE), (CAAE: 357807200.8.0000.0071, opinion number 4.186.699) and the Informed Consent Terms were signed by those responsible.

## CASE REPORT

Patient 1 was 14 years old, weight 40kg, and had severe AD for 13 years. Since 2012, he had been trying treatment with several immunosuppressive drugs, such as high-dose oral corticosteroids for 4 years, cyclosporine at maximum dose for 6 months, and methotrexate for 3 months, besides other therapeutic options, such as phototherapy, antihistamines, and frequent use of topical corticosteroids and moisturizers, including wet occlusive bandages.

Patient 2, his brother, was 16 years old, weight 43kg, and had severe AD since the age of 2 years. As of 2012, he had tried high-dose oral corticosteroids for 4 years and azathioprine for 6 months, as well as phototherapy, antihistamines, and the same topical treatments as his brother.

Both had delayed growth and pubertal development, which required them to replace growth hormone and testosterone. They also had recurrent skin infections and allergic conjunctivitis associated with AD. They stopped attending school due to socialization problems, since they suffered bullying and had poor school performance. They could not sleep, due to the constant itching of severe AD. Due to poor sleep, they also presented with irritability, attention *deficit*, and other situations that prevented them from having a normal life, with a minimum quality of life.

The mother could not report the exact dose of immunosuppressive medications used in the past by her children, and when they came to the first consultation, they were only using oral antihistamines, high potency topical corticosteroids, and moisturizers, disbelieving in treatments in general.

The dermatological examination showed extensive eczema affecting approximately 90% of skin, accompanied by very intense pruritus and xerosis, and a Scoring Atopic Dermatitis (SCORAD) of 99 for the 14-year-old, and 87.5 for the 16-year-old patients. SCORAD is a tool to assess severity of AD by scoring for signs and symptoms, and ranges from zero (no lesions and symptoms) to 103 (maximum). Atopic dermatitis is considered severe when score is above 50.^(^^9^^)^

With the disclosure of results with the monoclonal antibody dupilumab for individuals >12 years of age, and its approval in several countries, including Brazil, the medication was indicated.^(^^10^^)^ However, since the suggested dose for their weight (less than 60kg, ampoule of 200mg) was not yet available in Brazil (nor was there any forecast for it), we indicated the use of the adult dose.

Treatment was given on an outpatient basis. Their first dose of dupilumab 600mg subcutaneously was administered on January 31, 2020. They then started receiving 300mg every 2 weeks, as indicated for people ≥60kg. Since the first dose, they have already shown some improvement. Currently, after eight doses, the current SCORAD is 34.6 and 17.3 for the 14- and 16-year-old patients, respectively. Dermatological examination of eczema shows intense improvement, as well as xerosis and pruritus.

**Figure 1:**
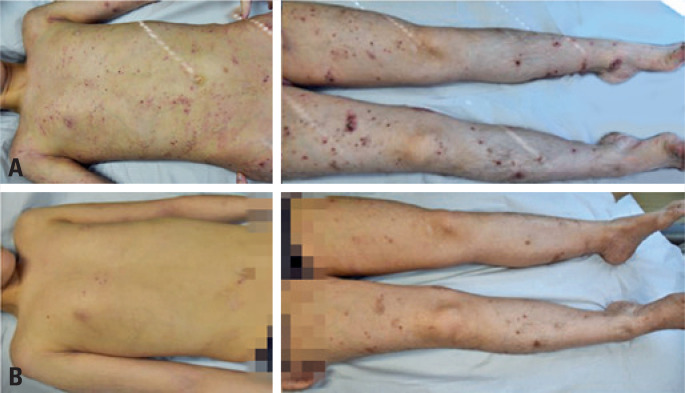
Frontal image of patient 1 before (A) and after eight treatment doses (B) with dupilumab

**Figure 2:**
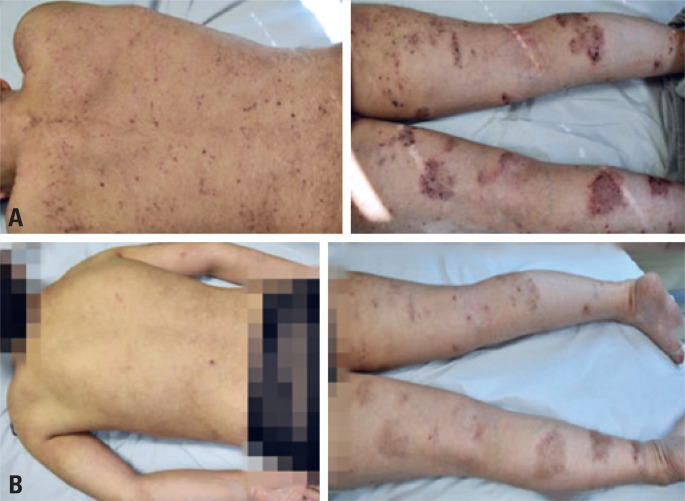
Dorsal image of patient 1 before (A) and after eight treatment doses (B) with dupilumab

Figures 1 and 2, and table 1 show the improvement obtained between the day of the first dose of treatment and after the eighth dose. The side effect identified only in patient 1, was worsening of conjunctivitis in the 3 days following the application.

**Table 1:** Evaluation of severity of atopic dermatitis using the Scoring Atopic Dermatitis before and after treatment with dupilumab

Medication	SCORAD of patient 1	SCORAD of patient 2
Oral antihistamine, high-potency topical corticosteroid, and moisturizer	99.0	87.5
After 8 injections of dupilumab, high-potency topical corticosteroid, and moisturizer	34.6	17.3

*SCORAD: Scoring* Atopic Dermatitis.

## DISCUSSION

Dupilumab is a fully humanized monoclonal antibody with direct action on the common alpha chain of interleukin (IL) receptors 4 and 13. These two cytokines are involved in the T2 immune response profile, inducing allergic sensitization, promoting atopic inflammation, and decreasing the skin barrier function and structure.^(^^11^^)^ The antibody inhibits the action of these cytokines and is associated with altered gene expression in AD lesions, improving their molecular signature.^(^^12^^)^ In a phase III clinical trial (NCT03054428) involving 251 adolescents with moderate to severe AD who were not controlled with topical treatment, it was noted that dupilumab improved the signs and symptoms of the disease, including pruritus, anxiety, depression, and quality of life. Skin infections were significantly less frequent in the treated group *versus* placebo. The two regimens tested, *i.e*., 200mg (≤60kg) or 300mg (≥60kg) subcutaneously every 2 weeks, or 300mg subcutaneously every 4 weeks for 16 weeks, were equally effective and safe. The most frequent side effects were injection site reactions and conjunctivitis.^(^^10^^)^ This same safety and efficacy profile had already been demonstrated in adults.^(^^11^^)^

We reported the case of two brothers with severe and poorly controlled AD, despite having received the most effective systemic treatments available in Brazil. They used corticosteroids and other immunosuppressants for years, which did not bring the desired control of AD. We believe growth delay is associated with long-term oral corticosteroid use. There are studies reporting that AD itself is associated with the production of prostaglandin E2 (PGE2) and platelet-activating factor, which alter osteoblasts and retard growth.^(^^13^^)^ They had continuous pruritus and widespread skin lesions, in addition to poor quality of life, with repercussions in their school, social, and psychological life. We were left with the choice of using immunosuppressants again or using dupilumab at a dose for patients ≥60kg, the only one available in the Brazilian market up to that moment, despite the fact they weighed roughly 40kg.

## CONCLUSION

We reported the first cases of use of dupilumab in Brazil in two adolescent siblings weighing approximately 40kg, with a dose of the drug for patients ≥60kg, due to its unavailability in the national market in the suggested dose. This unusual situation was due to the patients’ critical condition: prolonged history of severe atopic dermatitis with no control with previous immunosuppressive therapies, delayed growth and development, and very poor quality of life for them and their families. The use of this new drug class to control atopic dermatitis at the dose used by us proved to be very effective and safe for patients of this age group, with tolerable side effects and a high level of satisfaction for the adolescents and their family.
