# Supplementary material for: Efficacy and safety of dupilumab in two adolescents with severe atopic dermatitis
Source: Einstein (Sao Paulo). 2021 Apr 28;19:eRC6064. doi: 10.31744/einstein_journal/2021RC6064 (PMC8081439; doi:10.31744/einstein_journal/2021RC6064)
Supplement: Translation

# Translation: Eficácia e segurança do uso de dupilumabe em dois adolescentes com dermatite atópica grave

**Authors:** Mara Giavina-Bianchi, Pedro Giavina-Bianchi

**Affiliations:** 1 São PauloSP Brasil Dermatologista, São Paulo, SP, Brasil.; 2 São PauloSP Brasil Faculdade de Medicina, Universidade de São Paulo, São Paulo, SP, Brasil.

**Keywords:** Dermatite atópica/tratamento farmacológico, Conjuntivite alérgica, Imunossupressores, Índice de gravidade de doença, Agentes dermatológicos, Dupilumabe, Azatioprina, Adolescente, Eficácia, Segurança

## Abstract

Relatamos os casos de dois irmãos adolescentes com dermatite atópica grave e que, apesar de pesarem cerca de 40kg, apresentaram boa resposta ao dupilumabe com a dose *off-label* para indivíduos com 12 anos e peso de 60kg. Ambos já tinham usado ciclosporina, azatioprina, metotrexato e corticoide oral por longos períodos, acrescidos de tratamentos tópicos sem controle adequado da doença. As lesões cutâneas eram constantes e disseminadas, e os irmãos apresentavam infeções de pele frequentes e qualidade de vida muito ruim, com inúmeras consequências físicas e psicossociais, como o abandono da atividade escolar pelo prurido intenso, pela aparência e pelo *bullying* sofrido. Apresentavam também retardo de crescimento e de desenvolvimento. Em 2018, o dupilumabe, um agente imunobiológico, foi aprovado para o tratamento de dermatite atópica moderada a severa para adultos e, em 2019, ampliado para faixa etária de 12 a 17 anos. Embora já tivesse a aprovação da Agência Nacional de Vigilância Sanitária no Brasil, a apresentação de 200mg (indicada para o peso dos pacientes) não estava disponível, sem previsão de chegada. Assim, pesando os riscos e benefícios da situação de ambos, optamos por tratá-los com dose de adulto (ataque de 600mg por via subcutânea e 300mg por via subcutânea a cada 2 semanas) apesar do baixo peso. Até o momento, eles realizaram oito aplicações, apresentando importante melhora da doença e da qualidade de vida. Não houve efeitos adversos importantes – apenas a piora da conjuntivite alérgica em um deles. Os pacientes e sua família estão muito satisfeitos, e nós avaliamos que a terapia está sendo bem-sucedida.

## INTRODUÇÃO

A dermatite atópica (DA) é uma doença inflamatória crônica da pele, recorrente, extremamente pruriginosa, composta por lesões maculopapulares eritematosas ou vesiculares, descamativas, acompanhadas de ressecamento, crostas e/ou liquenificação. A infecção secundária por vírus ou bactérias é comum. A prevalência é de 15% em crianças e 5% em adultos e está aumentando.^(^^1^^–^^3^^)^

Viver com DA pode ser difícil, especialmente para quem necessita de tratamento sistêmico prolongado, porque os medicamentos podem ter importante toxicidade. A coceira intensa e as lesões cutâneas podem causar distúrbios do sono, ansiedade, depressão e baixa autoestima, comprometendo a qualidade de vida dos pacientes e familiares.^(^^4^^)^

A patogênese da DA inclui alteração da barreira cutânea, podendo estar associada a mutações do gene da filagrina, aumento da colonização por *Staphylococcus aureus* e resposta imune Th2 exacerbada, com sensibilização a alérgenos, níveis elevados de imunoglobulina E (IgE) e eosinofilia no sangue. A ciclosporina, o micofenolato de mofetila, a azatioprina e o metotrexato são os tratamentos imunossupressores mais utilizados.^(^^4^^–^^7^^)^ Novas terapias, como o dupilumabe e os inibidores da enzima Janus kinase (JAK), baseadas na patogênese da DA, mais eficazes e menos prejudiciais, estão sendo desenvolvidas e vem alterando a abordagem da DA moderada a grave.^(^^5^^)^ Atualmente, o dupilumabe está aprovado para os casos de DA moderada a grave, asma grave e rinossinusite crônica com polipose nasal em crianças a partir de 6 anos, adolescentes a partir dos 12 anos e adultos a partir dos 18 anos, respectivamente.^(^^8^^)^

O relato a seguir descreve a grande melhora obtida no tratamento da DA grave nos dois primeiros adolescentes com menos de 60kg tratados no país usando-se o dupilumabe.

O estudo foi aprovado pelo Comitê de Ética em Pesquisa do Hospital Israelita Albert Einstein (HIAE), (CAAE: 357807200.8.0000.0071. número do parecer 4.186.699) e os Termos de Consentimento Livre Esclarecido foram assinados pelos responsáveis.

## RELATO DE CASO

O paciente 1 tinha 14 anos, 40kg e apresentava DA grave há 13 anos. Desde 2012, tentava tratamento com diversos imunossupressores, como corticoide via oral em altas doses por 4 anos, ciclosporina em dose máxima por 6 meses, e metotrexato por 3 meses, além de outras opções terapêuticas, como fototerapia, anti-histamínicos e uso frequente de corticosteroides tópicos e hidratantes, inclusive com bandagens oclusivas molhadas.

O paciente 2, seu irmão, tinha 16 anos, 43kg e apresentava DA grave desde os 2 anos. Desde 2012, tentou tratamento com corticoide oral em altas doses por 4 anos e azatioprina por 6 meses, além de fototerapia, anti-histamínicos e os mesmo tratamentos tópicos do irmão.

Ambos tinham retardo do crescimento e do desenvolvimento puberal, que os obrigava a repor hormônio do crescimento e testosterona. Apresentam também infecções de pele recorrentes e conjuntivite alérgica associada à DA. Deixaram de frequentar a escola por problemas de socialização, pois sofriam “*bullying*” e apresentavam baixo desempenho escolar, uma vez que não conseguiam dormir, devido ao prurido constante da DA grave. Devido ao sono ruim, apresentavam irritabilidade, *deficit* de atenção e outras situações que os impedem de ter uma vida normal, com um mínimo de qualidade de vida.

A mãe não sabia relatar a dose exata das medicações imunossupressoras utilizadas no passado pelos filhos, e, quando eles vieram à primeira consulta, usavam somente anti-histamínico via oral, corticoides tópicos de alta potência e hidratantes, descrente dos tratamentos em geral.

Ao exame dermatológico, apresentavam quadro de eczema extenso, acometendo cerca de 90% do tegumento, acompanhado de prurido e xerose muito intensos, e pontuação no *Scoring* *Atopic Dermatitis* (SCORAD) de 99 para o paciente de 14 anos e 87,5 no de 16 anos. O SCORAD é uma ferramenta para avaliar a gravidade da DA por meio de pontuação para sinais e sintomas e pode variar de zero (sem lesões e sintomas) a 103 (máximo). Acima de 50, a DA é considerada grave.^(^^9^^)^

Com a divulgação dos resultados com o anticorpo monoclonal dupilumabe para indivíduos >12 anos, e sua a aprovação em diversos países, Brasil inclusive, foi indicada a medicação.^(^^10^^)^ No entanto, como a dose sugerida para o peso deles (menor que 60kg, ampola de 200mg) não estava disponível ainda no Brasil (e nem havia previsão para tal), indicamos o uso da dose de adulto.

O tratamento foi feito em ambulatório. A primeira dose de dupilumabe 600mg por via subcutânea foi administrada em 31 de janeiro de 2020. Depois, passaram a receber 300mg a cada 2 semanas, como indicado para pessoas ≥60kg. Desde a primeira tomada, eles já apresentaram alguma melhora. Atualmente, após oito doses, o SCORAD atual é de 34,6 e 17,3 para os pacientes de 14 e 16 anos, respectivamente. O exame dermatológico do eczema exibe intensa melhora, assim como xerose e prurido.

As figuras 1 e 2 e a tabela 1 demonstram a melhora obtida entre o dia da primeira dose do tratamento e após a oitava tomada. O efeito colateral identificado foi piora da conjuntivite nos 3 dias que se seguiram à aplicação, somente no paciente 1.

**Figura 1:** Imagem frontal do paciente 1 antes (A) e após oito doses de tratamento (B) com dupilumabe

**Figura 2:** Imagem dorsal do paciente 1 antes (A) e após oito doses de tratamento (B) com dupilumabe

**Tabela 1:** Avaliação da gravidade da dermatite atópica por meio do *Scoring Atopic Dermatitis* antes e após o tratamento com dupilumabe

Medicamento	SCORAD do paciente 1	SCORAD do paciente 2
Anti-histamínico via oral, corticoide tópico de alta potência e hidratante	99,0	87,5
Após 8 tomadas de dupilumabe, corticoide tópico de alta potência e hidratante	34,6	17,3

SCORAD: *Score for Atopic Dermatitis.*

## DISCUSSÃO

O dupilumabe é um anticorpo monoclonal totalmente humanizado, com ação direta sobre a cadeia alfa, comum dos receptores de interleucina (IL) 4 e 13. Essas duas citocinas estão envolvidas no perfil de resposta imune T2, induzindo sensibilização alérgica, promovendo a inflamação atópica e diminuindo a função e a estrutura da barreira cutânea.^(^^11^^)^ O anticorpo inibe a ação dessas citocinas e está associado com alteração da expressão de genes em lesões de DA, melhorando sua assinatura molecular.^(^^12^^)^ Em ensaio clínico fase III (NCT03054428), envolvendo 251 adolescentes com DA moderada a grave que não estavam controlados com tratamento tópico, observou-se que o dupilumabe melhorou os sinais e os sintomas da doença, incluindo prurido, ansiedade, depressão e qualidade de vida. As infecções de pele foram significativamente menos frequentes no grupo tratado em relação ao placebo. Os dois regimes testados, 200mg (≤60kg) ou 300mg (≥60kg) subcutâneo a cada 2 semanas ou 300mg subcutâneo a cada 4 semanas por 16 semanas, foram igualmente eficazes e seguros. Os efeitos colaterais mais frequentes foram reações no local da injeção e conjuntivite.^(^^10^^)^ Esse mesmo perfil de segurança e eficácia já tinha sido demonstrado em adultos.^(^^11^^)^

Expusemos o caso de dois irmãos com DA grave e mal controlada, apesar de terem feito os tratamentos sistêmicos mais eficazes até o momento disponíveis no Brasil. Fizeram uso de corticoide e outros imunossupressores por anos, os quais não trouxeram o controle desejado da DA. Pensamos que o retardo de crescimento esteja associado ao uso do corticoide oral por longo período. Há estudos relatando que a própria DA se asso cia à produção de prostaglandina E2 (PGE2) e fator de ativação plaquetária, que alteraram os osteoblastos e retardam o crescimento.^(^^13^^)^ Apresentavam prurido e lesões cutâneas disseminadas continuadamente, além de importante comprometimento da qualidade de vida, com repercussão na vida escolar, social e psicológica. Restavam-nos as opções de voltar a usar imunossupressores ou usar o dupilumabe na dose de pacientes ≥60kg, única disponível no mercado brasileiro até aquele momento, apesar de eles pesarem em torno de 40kg.

## CONCLUSÃO

Relatamos os primeiros casos do uso no Brasil do dupilumabe em dois irmãos adolescentes com cerca de 40kg, com dose de medicamento para pacientes ≥60kg, devido à indisponibilidade no mercado nacional na dose sugerida. Essa situação incomum deveu-se ao estado crítico em que os pacientes se encontravam: história prolongada de dermatite atópica grave sem controle com terapias imunossupressoras prévias, retardo de crescimento e desenvolvimento e baixíssima qualidade de vida deles e dos familiares. O uso dessa nova classe de droga para controle da dermatite atópica na dose utilizada por nós se revelou muito eficaz e segura para pacientes dessa faixa etária, com efeitos colaterais toleráveis e alta satisfação para os adolescentes e sua família.
